# Inhibitory control in children with tic disorder: aberrant fronto-parietal network activity and connectivity

**DOI:** 10.1093/braincomms/fcab067

**Published:** 2021-04-09

**Authors:** Joseph Jurgiel, Makoto Miyakoshi, Andrea Dillon, John Piacentini, Scott Makeig, Sandra K Loo

**Affiliations:** 1 Semel Institute for Neuroscience and Human Behavior, University of California, Los Angeles, Los Angeles, CA 90095, USA; 2 Swartz Center for Neural Computation, University of California, San Diego, La Jolla, CA 92093, USA

**Keywords:** electroencephalography, neural oscillations, effective connectivity, movement disorders, Tourette syndrome

## Abstract

Chronic tic disorders, including Tourette syndrome, are typically thought to have deficits in cognitive inhibition and top down cognitive control due to the frequent and repetitive occurrence of tics, yet studies reporting task performance results have been equivocal. Despite similar behavioural performance, individuals with chronic tic disorder have exhibited aberrant patterns of neural activation in multiple frontal and parietal regions relative to healthy controls during inhibitory control paradigms. In addition to these top down attentional control regions, widespread alterations in brain activity across multiple neural networks have been reported. There is a dearth, however, of studies examining event-related connectivity during cognitive inhibitory paradigms among affected individuals. The goal of this study was to characterize neural oscillatory activity and effective connectivity, using a case–control design, among children with and without chronic tic disorder during performance of a cognitive inhibition task. Electroencephalogram data were recorded in a cohort of children aged 8–12 years old (60 with chronic tic disorder, 35 typically developing controls) while they performed a flanker task. While task accuracy did not differ by diagnosis, children with chronic tic disorder displayed significant cortical source-level, event-related spectral power differences during incongruent flanker trials, which required inhibitory control. Specifically, attenuated broad band oscillatory power modulation within the anterior cingulate cortex was observed relative to controls. Whole brain effective connectivity analyses indicated that children with chronic tic disorder exhibit greater information flow between the anterior cingulate and other fronto-parietal network hubs (midcingulate cortex and precuneus) relative to controls, who instead showed stronger connectivity between central and posterior nodes. Spectral power within the anterior cingulate was not significantly correlated with any connectivity edges, suggesting lower power and higher connectivity are independent (versus resultant) neural mechanisms. Significant correlations between clinical features, task performance and anterior cingulate spectral power and connectivity suggest this region is associated with tic impairment (*r* = −0.31, *P* = 0.03) and flanker task incongruent trial accuracy (*r*’s = −0.27 to −0.42, *P*’s = 0.0008–0.04). Attenuated activation of the anterior cingulate along with dysregulated information flow between and among nodes within the fronto-parietal attention network may be neural adaptations that result from frequent engagement of neural pathways needed for inhibitory control in chronic tic disorder.

## Teaser

Jurgiel *et al*. examined cortical source-resolved neural dynamics occurring during a flanker paradigm among children with and without chronic tic disorders. Attenuated spectral power within the anterior cingulate as well as atypical fronto-parietal network communication was apparent in children with tic disorders.

## Graphical Abstract

**Graphical Abstract:**
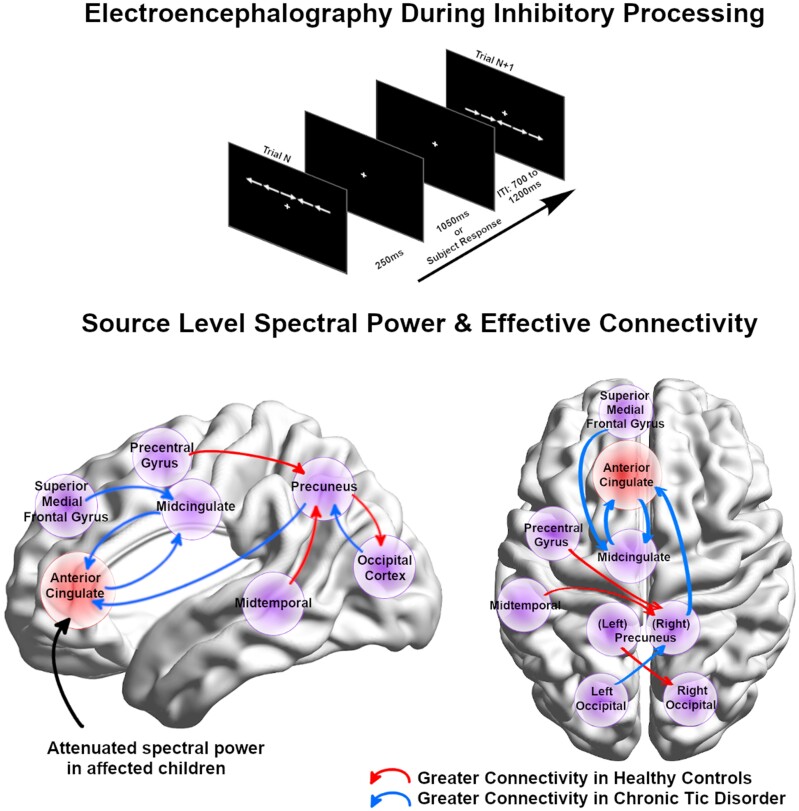

## Introduction

Chronic tic disorders (CTDs), including Tourette syndrome, are characterized by sudden, involuntary, and recurrent movements or vocalizations, referred to as tics. Given the frequent and repetitive occurrence of tics, it has been hypothesized that this atypical behaviour is due, at least in large part, to deficits in motor inhibition and top down cognitive control, as these processes are thought to mediate the selection of appropriate actions and behaviours. Numerous tasks have been used to evaluate the presence of inhibitory control deficits in CTD, however, results have been equivocal. For example, previous studies using different cognitive control paradigms have reported no differences in task performance between individuals with CTD and healthy controls.^1^^,^^2^ In contrast, others have observed worse performance^3^^,^^4^ as well as better performance in CTD when compared to controls,^5^^,^^6^ leading to uncertainty as to the scope of potential deficits in cognitive control. A recent meta-analysis found a moderate effect (Cohen’s *d* = 0.33) of general inhibitory deficits in CTD relative to typically developing controls, however, the effect size varied by task with commonly used inhibitory paradigms (Stop Signal, Flanker, and Go/No-go tasks) being not significantly different between diagnostic groups.^7^ Findings of similar or better performance in individuals with CTD have been attributed to compensatory mechanisms developed from repeated voluntary tic suppression.^5^^,^^6^

The Eriksen flanker task is a common cognitive paradigm for measuring inhibitory control where the subject must quickly indicate the direction of a central arrow flanked by arrows pointing in a congruent or conflicting direction.^8^ Among healthy adults, activation of the anterior cingulate cortex (ACC) occurs during incongruent flanker trials,^9^ which is thought to play an important role in monitoring and/or resolving such conflict. The anterior insular cortex, precentral gyrus, intraparietal sulcus and bilateral occipital cortices have also been reported to be involved in inhibitory control.^10^^,^^11^ In contrast, typically developing children demonstrate developmental differences during inhibitory processing with activation of more central and posterior regions (premotor cortex, superior temporal gyrus, bilateral parietal and occipital cortices) relative to inferior and medial frontal gyri and ACC, potentially suggesting the inhibitory network is slower to develop in frontal areas.^12^^,^^13^

Despite similar inhibitory control task performance, individuals with CTD have exhibited aberrant patterns of neural activation in the supplemental motor area, anterior cingulate, sensorimotor, inferior frontal and inferior parietal cortices relative to healthy controls.^14–17^ Several of these same brain areas have been implicated in other experimental conditions such as resting state, voluntary movement, and voluntary tic suppression.^18–23^ While regional activation analyses are valuable for pathophysiological hypothesis testing, whole brain analysis using effective connectivity may be particularly useful yet underexplored in CTD. Prior studies and meta-analyses have suggested that alterations in brain activity are present across multiple neural systems^24–26^ and thus identification of mechanistic pathways of dysfunction may reveal greater insights than higher or lower neural activation.

Several studies examining resting state connectivity within CTDs have reported widespread atypical network connectivity that vary according to development. For example, paediatric studies have reported a trend of hypo- and immature connectivity among circuitry including fronto-parietal, posterior, and default-mode networks.^27–29^ In contrast, adult studies suggest increased structural^30^^,^^31^ and functional connectivity with decreased functional hub count^32^ within cortico-basal ganglia circuitry and urge-tic networks, which are positive correlated with tic severity.^31–33^ Such differences may be due to the heterogenous and developmental nature of childhood CTD compared to the more stable state of adult CTD, as well as compensatory mechanisms that develop over time through learned self-regulation.^6^ Consistent with this are recent findings that functional connections that best discriminate individuals with CTD from controls are age-specific, with default mode and fronto-parietal connections providing the best discrimination among children and salience, somatomotor, and default mode features best discriminating adults with CTD.^34^ Collectively, both biregional and whole-brain connectivity patterns among individuals with CTD are aberrant with the nature of deficit being across development.

Resting state paradigms, however, do not clarify the effect of differential neural connectivity on cognitive processes, nor whether particular connectivity patterns are adaptive or disruptive features of the disorder, an important factor for evaluating developmental trajectories. Across brain imaging modalities, aberrant connectivity during self-paced finger movements,^35^ waiting motor impulsivity^36^ and face perception paradigms^37^ have been noted among individuals with CTD. There is a notable dearth, however, of studies examining event-related connectivity during inhibitory control paradigms among individuals with CTD, particularly those using electroencephalography (EEG), which can reveal network activities with millisecond time resolution. There are two EEG-based studies that reported higher frontomesial and fronto-motor functional connectivity during voluntary tic suppression^38^^,^^39^ as well as during a Go/No-Go task (compared to controls).^38^ However, both studies were small (*N* = 9–10 children with CTD, 10 controls) and utilized a low number (10–19) of EEG channels. In addition, the studies examined scalp-level connectivity, where there is often concern of spurious connectivity measurements due to volume conduction between nearby scalp electrodes.^40^ We have recently developed an algorithm for estimating cortical-source level, effective connectivity based on high density EEG^20^^,^^41^ and will apply that approach here to gain insight into putative atypical connectivity patterns within a sample of children with CTD while performing an inhibitory task. We hypothesized that children with CTD would not differ from typically developing controls in flanker task accuracy based on prior meta-analytic findings.^7^ We did, however, hypothesize that the diagnostic groups would diverge in terms of activation and connectivity patterns of top down attentional control and inhibitory networks within frontal and parietal cortices.

## Materials and methods

### Sample

The case–control study sample consisted of 95 children [60 with chronic tic disorder (CTD), 35 healthy controls (HC)], aged 8–12 years old. Participants were recruited through community advertisements, internet postings, and from an academic medical center anxiety and tic disorder clinic between 2013 and 2019. Verbal and written explanations of study criteria were provided to participants and their parents, and written parent permission/assent were obtained prior to study participation. All study procedures and consents were approved by the local Institutional Review Board.

### Procedure

All participants participated in a single experimental session that lasted approximately 2–3 h, during which time diagnostic interviews, cognitive testing, and EEG recording were administered. Psychiatric diagnoses were determined using a semi-structured diagnostic interview, the Anxiety Disorder Interview Schedule, Child Version (ADIS)^42^ modified to assess chronic tic disorders. Diagnostic interviews were administered either by supervised graduate level psychologists or directly administered by a licensed psychologist, who confirmed the presence of DSM-5 psychiatric diagnoses. The ADIS was supplemented by the clinician-administered Yale Global Tic Severity Scale (YGTSS),^43^ Child Yale-Brown Obsessive-Compulsive Scale (CYBOCS),^44^ and Strengths and Weaknesses of ADHD symptoms and Normal behaviour (SWAN) scale.^45^ Estimated intelligence (IQ) was assessed using the Wechsler Abbreviated Scale of Intelligence (WASI).^46^

Participants were included in the study if they met the following criteria: (i) male or female aged 8–12 years; (ii) resided with their primary caretaker for at least 6 months prior to consent; (iii) both participant and guardian(s) were able to complete all study measures in English; and (iv) capable of completing all required study procedures (as determined by psychologist). Individuals with CTD were required to have a primary DSM-5 diagnosis of Persistent Motor Tic Disorder, Persistent Vocal Tic Disorder, or Tourette Disorder as diagnosed by ADIS and confirmed by diagnostic interview, as well as YGTSS ≥ 15 at baseline. Individuals with CTD were excluded from participation if they had a history of any of the following: (i) head injury resulting in concussion; (ii) diagnoses of autism, major depression, bipolar disorder, panic disorder, or psychosis; (iii) estimated Full Scale IQ < 80; or (iv) YGTSS < 15 (CTD only). Individuals taking stimulant medication for comorbid ADHD discontinued use for 24 h prior to their visit. Other psychotropic medications were included as covariates of no interest in analyses. Healthy controls were excluded if they had any major Axis I diagnosis or were on a psychoactive medication.

### Experimental task

**Figure 1:**
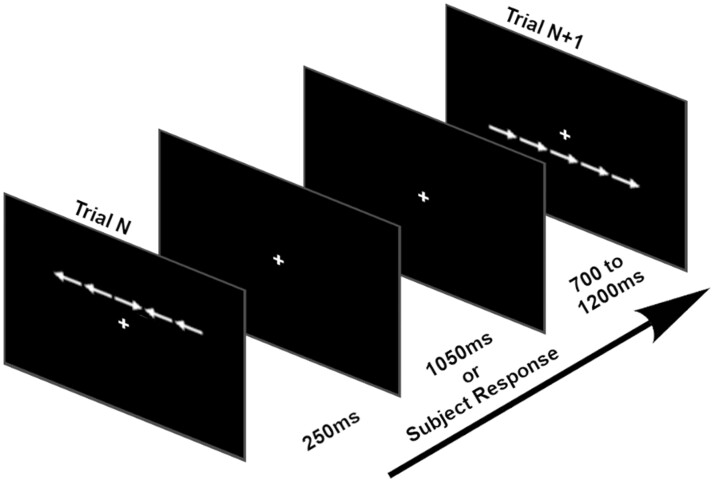
**Modified Eriksen flanker task.** The figure illustrates an incongruent flanker trial (in which the middle arrow points in a different direction than the flanking arrows) followed by a congruent flanker trial (in which all arrows point in the same direction).

Participants performed a modified Eriksen flanker task^8^ while EEG was recorded (Fig. 1). Each trial consisted of five white arrows appearing horizontally across a black screen either above or below a centralized, static white fixation cross. Each arrow spanned 1.2° × 1.2° visual angle with 0.6° spacing between arrows. The arrows remained on the screen for 250 ms before disappearing. Following subject response or trial expiration due to lack of response after 1300 ms from stimulus onset, a jittered 700–1200 ms intertrial interval succeeded the trial until the next set of arrows appeared. The arrows could appear in congruent orientation, where all arrows pointed in the same direction, or incongruent orientation, where the central arrow pointed in the opposite direction of the side flanking arrows. The subject used a computer mouse with their right hand to press the left or right mouse button to select the respective direction of the central arrow. The task consisted of a high number of incongruent trials (*n* = 144) versus congruent trials (*n* = 72) to increase the inhibitory control required, which was a primary focus of the study. Trials were presented in random order and the task lasted approximately 7 min; dependent variables were percent accuracy, reaction time (on correct trials only) and reaction time variability.

### EEG recording and processing

EEG was recorded using a 128 Hydrocel electrode net in an extended international 10–10 configuration (Electrical Geodesics Incorporated). Electrode scalp coordinates were transcribed through Polhemus, Inc. digitizer software, using the nasion and preauricular notches as anatomical reference points. Data were sampled at 1000 Hz, referenced to Cz, and electrode impedances were lower than 50 kΩ (per manufacturer recommendation). Task event markers from E-Prime software were merged with raw EEG signals using Lab Streaming Layer (LSL, https://github.com/sccn/labstreaminglayer).

Data cleaning and processing was performed using EEGLAB.^47^ Spectral power analyses utilized data that were down sampled to 250 Hz and filtered using a 0.5–55 Hz bandpass filter. To remove channel artefacts, Artefact Subspace Reconstruction (ASR)^48–51^ was employed via EEGLAB plug-in *clean_rawdata()*, which removed channels with over 5 s of flat signal as well as those poorly correlated (*r* < 0.85) with adjacent channels. ASR additionally helped to remove and interpolate non-stationary high amplitude bursts. Scalp signals were then decomposed into independent source level activations, also known as independent components (ICs), using adaptive mixture independent component analysis (AMICA).^52–54^ This was performed within EEGLAB on the full EEG dataset, using approximately 525,000 data points (35 min of data sampled at 250 Hz) for 128-channel decomposition. IC rejection was performed using the EEGLAB plug-in ICLabel,^55^ an algorithm trained to detect neural versus non-neural IC activations. ICs were rejected if the brain was not the highest probability source. Dipole locations of source activations were subsequently estimated using Fieldtrip.^56^ For the effective connectivity analysis, EEG data were down sampled to 100 Hz to reduce model complexity and the potential influence of line noise. Each subject was also restricted to their top 10 ICs based on variance accounted for. This facilitated proper fitting of a multivariate autoregressive (MVAR) model using a sufficient data point ratio as proposed in Korzeniewska et al.^57^ based on the equality:

$$\frac{K^{2}\left(p+1\right)}{\mathrm{NsNt}}\leq 0.1$$

where *K* denotes the number of ICs, *p* is the model order, *N*_s_ is the number of samples in the sliding window, and *N*_t_ is the number of trials. This equation was modified slightly (using *K*^2^ instead of *K*) based on recommendations from the Source Information Flow Toolbox (SIFT).^58^

To perform event-related analyses, the preprocessed source-level data were epoched (from −2000 ms to 2000 ms around stimulus) into three trial types: congruent trials with correct responses, incongruent trials with correct responses and incongruent trials with incorrect responses. Congruent trials with incorrect responses were excluded due to sparsity.

### Event-related spectral power

**Figure 2:**
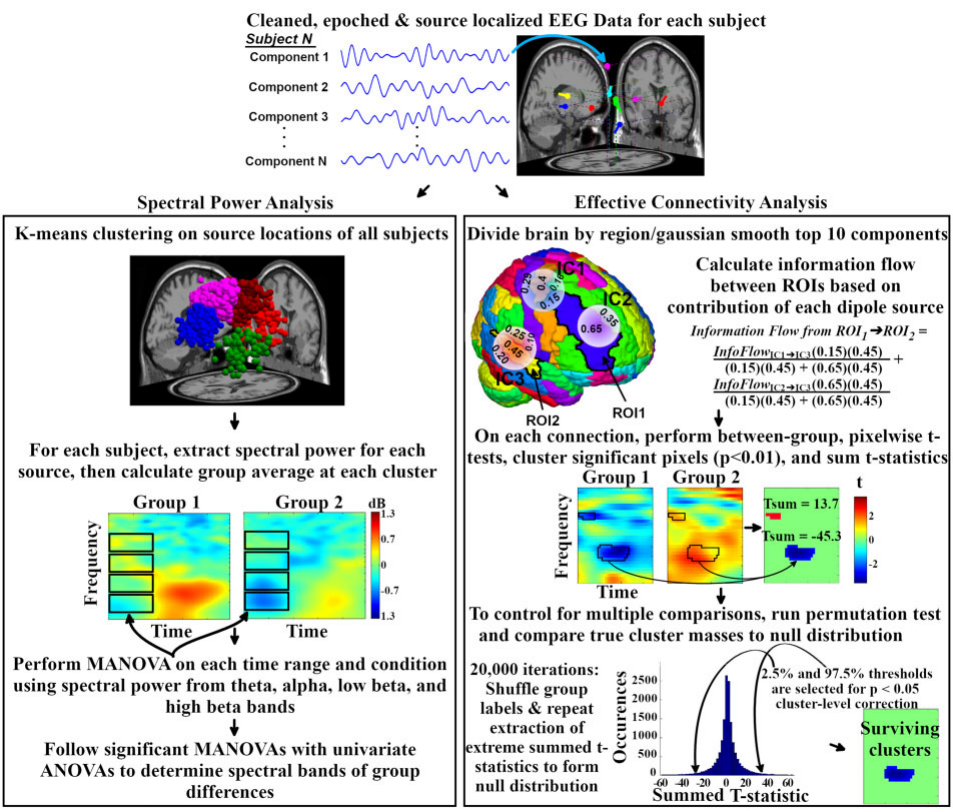
**Summary of EEG analysis methods for spectral power and effective connectivity.** ANOVA, analysis of variance; IC, independent component; MANOVA, multivariate analysis of variance; ROI, region of interest.

Event-related spectral perturbation (ERSP) is a time-frequency analysis method that measures event-related changes, relative to baseline, in spectral power evoked by a stimulus (see Fig. 2). To calculate ERSP, a sliding window of 1-second in length was applied across each epoch in 25 ms steps from which the average amplitude spectra of each frequency interval was calculated for each window using a Morlet kernel and the EEGLAB function *newtimef()*. These values were first averaged across trials, then baseline normalized using average power between −550 and −50 ms prior to stimulus, and converted into decibel (dB) values using 10*log_10_(X), producing time-frequency plots of log-scale spectral power. This time selection for baseline ensured that prior trial response phenomena were not included. Average ERSP values in frequency bands (Theta [4–7 Hz], Alpha [8–12 Hz], Low Beta [13–20 Hz], High Beta [20–30 Hz]) and time ranges related to stimulus presentation (0–200 ms) and conflict resolution/response preparation (250–600 ms) were extracted.

Dipoles were spatially grouped at the study level using *k*-means clustering weighted by dipole location (dimension, 3; weight, 10), event-related potential (dimension, 4; weight, 1), ERSP (dimension, 4; weight, 1) and scalp map (dimension, 6; weight, 1) to create a 12-cluster solution. Clusters that contained at least 70% of unique subjects were included in the spectral power analysis.

### Connectivity analysis

To examine network information flow, source-level effective connectivity was measured in the form of renormalized partial directed coherence (rPDC), a frequency-domain measurement of causal (directed) information flow between multivariate time series (see Fig. 2).^59^ This was done using the EEGLAB plugin *groupSIFT*,^20^ which utilizes the EEGLAB-compatible SIFT plug-in to calculate subject-level multivariate effective connectivity and perform group-level analyses. For each subject, rPDC was calculated between ICs using a MVAR model fitted from the Vieira-Morf algorithm. A 1-second sliding window was applied to the epoched data in 20 ms steps, along with 30 log-scaled frequencies from 2 to 49 Hz. This resulted in a 150 timepoint × 30 frequency matrix of rPDC values for each connection.

A challenge of source-space analyses is the variability in the locations of estimated dipole locations for each subject, which makes group-level comparisons of connectivity between regions difficult. To resolve this issue, *groupSIFT* utilizes a 3-D Gaussian kernel to ‘smooth’ dipoles from single points into probabilistic dipole densities. The full width at half maximum was set to 20 mm and the Gaussian was truncated to 3σ, resulting in a density radius of 25.5 mm. The automated anatomical labelling (AAL) atlas,^60^ customized to integrate non-cortical regions into upper and lower basal regions to avoid misleading use of subcortical regions as EEG sources, was then referenced to segment a brain model into 76 regions of interest (ROI). From this, each subject maintained a four-dimensional matrix of size 76 (region, leaders) × 76 (region, followers) × 30 (frequency) × 150 (time). Distributed dipole density for all subjects were placed in this voxelized brain model, and ROIs which contained overlapping smoothed dipoles for at least 70% of unique subjects were included in the analysis.

### Correction for multiple comparisons

For each connection in the whole-brain connectivity analysis, pairwise *t*-tests between diagnostic groups were performed on each rPDC time-frequency plot at the pixel level and masked at *P* < 0.01 significance. Groups of neighbouring pixels with surviving *t*-statistics were combined and summed to ‘*t*-statistic cluster masses’, representing time-frequency ranges of significant group differences. To correct for multiple comparisons, cluster-level correction^61^^,^^62^ was implemented for control of familywise error rate (FWER) using a non-parametric permutation test (*N* = 10,000) by shuffling diagnostic group labels of rPDC matrices. For each iteration, pairwise *t*-tests, significance thresholding and *t*-statistic cluster generation were repeated using the prior method. Observing across all graph edges, the second largest negative and postive *t*-statistic cluster masses were stored to form a surrogate null distribution. True-data cluster masses were then compared to the surrogate null distribution using a one-tail *P* < 0.05 significance threshold to obtain across-edge corrected results. This implementation of cluster-level correction ensured that the number of false discoveries did not exceed a chosen value of *u = 1*, with at least 95% confidence.^61^

In order to examine potential relationships between connectivity and spectral power findings, separate ROI analyses were run on regions with significant spectral power findings. This ROI analysis was achieved using a similar statistical procedure as the whole-brain analysis, except that the surrogate distribution was instead created from the largest cluster mass from each permutation/iteration of the single graph edge of interest (rather than across all edges). This resulted in within-edge control of FWER.

### Statistical analysis

All analyses were run in the R programming environment^63^ using customized scripts. In summary, we used the following analysis path for obtaining and interpreting results. First, spectral power calculations were performed on *k*-means clustered sources to examine localized group differences in spectral power in pre-selected time and frequency ranges. To aide in functional interpretation, Pearson partial correlations (controlling for age) were then run between significant spectral power findings and clinical metrics (YGTSS scores) along with task performance. Next, whole-brain effective connectivity was examined along with supplemental ROI connectivity analyses on any regions with significant spectral power findings. Pearson partial correlations were run between significant connectivity findings and task performance, and with any corresponding spectral power differences to investigate potential compensatory relationships between local power and regional connectivity. Previous EEG analyses among children with and without CTD indicated large effect sizes in brain measurements during voluntary movement.^20^ Although the tasks differ across studies, our current sample size (*N* = 95) had sufficient power (>80%) to detect an alpha of 0.05 with a medium effect size (*f* = 0.20).^64^

Flanker task behavioural data were tested for diagnostic group differences using analysis of variance (ANOVA) for the dependent variables (accuracy, reaction time, reaction time variability) from congruent and incongruent trials separately. Effects of covariates (gender, age and ADHD/OCD comorbidity) on significant group differences were tested by re-running ANCOVAs with each covariate. To reduce dimensionality and type 1 error rate within the EEG spectral power analyses, multivariate analysis of variance (MANOVA) tests were used to examine significant group differences in spectral power. MANOVAs were run for each trial type, source cluster, and time period, with theta, alpha, low beta, and high beta band spectral power as dependent variables. MANOVAs with significant diagnostic group differences (*P* < 0.05) were followed by univariate ANOVAs to test the effect of diagnostic group on the spectral power of each frequency band. Effects of covariates (gender, age and ADHD/OCD comorbidity) on significant group differences were tested by re-running ANCOVAs with each covariate. Statistical procedures to control type 1 error for the connectivity analysis were performed in the *groupSIFT* toolbox described prior.

### Data availability

Anonymized data that support the findings of this study are available upon request.

## Results

Participants in the HC (*N* = 35) and CTD (*N* = 60) groups were well matched in age (HC = 9.6 ± 1.4, CTD = 10.0 ± 1.4, *t* = −1.2, *P* = 0.25). Estimated intelligence (IQ) was above the average range in both groups but modestly lower in the CTD group (HC = 115 ± 15, CTD = 110 ± 13, *t* = 2.1, *P* = 0.04), which has been reported for other clinical samples.^65^ Gender ratio differed, as the HC group was 46% male and the CTD group was 80% male (χ^2^ = 10.3, *P* = 0.001). To account for this imbalance, significant spectral power results were re-run using gender and IQ as covariates. The CTD group had an average YGTSS Total score of 27 ± 8.6, suggesting moderate clinical impairment. Within the CTD group, 10 had comorbid OCD, 15 had comorbid ADHD, and nine had both OCD and ADHD comorbidities, which is similar to rates of comorbidity for ADHD and/or OCD in other samples.^66^ A total of 10 participants were on non-stimulant psychotropic medication and one patient with CTD was taking a stimulant, which was discontinued for 24 h prior to their visit.

### Flanker performance

Significant diagnostic group differences on behavioural performance measures in reaction time for congruent and incongruent trials emerged, reflecting faster reaction times in the CTD group relative to controls (see Table 1). Accuracy and reaction time variability for both trial types were not significantly different across groups. When controlling for covariates, the congruent trial reaction time differences remained significant, while the incongruent trial reaction time differences continued to be significant when controlling for age and IQ, but not gender, OCD or ADHD symptoms.

**Table 1:** Flanker task performance and EEG spectral power differences between tic disorder group and controls

Performance	HC (M, SD)	CTD (M, SD)	*F*(1, 93)	With covariates				
				*F*_Gender_	*F*_Age_	*F*_IQ_	*F*_CYBOCS_	*F*_SWAN_
**Performance F(1,93)**								
Congruent trials								
Accuracy (%)	78 (18)	75 (16)	0.76					
Reaction time (ms)	564 (91)	507 (78)	10.70**	6.75*	9.11**	9.54**	6.37*	6.34*
Reaction time SD (ms)	141 (42)	129 (36)	2.00					
Incongruent trials								
Accuracy (%)	65 (19)	59 (16)	2.79					
Reaction time (ms)	606 (135)	541 (112)	6.35*	3.00	5.46*	4.42*	3.24	1.81
Reaction time SD (ms)	117 (15)	110 (13)	0.00					
**Spectral power (in dB) F(1,72)**								
Anterior cingulate, 0–200 ms								
Theta (4–7 Hz)	0.00 (1.06)	−0.42 (0.71)	4.21*	2.76	5.04*	5.09*	4.13*	3.79
Alpha (8–12 Hz)	0.30 (0.82)	−0.26 (0.67)	10.11**	7.65**	9.52**	8.21**	4.52*	4.68*
Low beta (13–20 Hz)	0.05 (0.47)	−0.11 (0.53)	1.64					
High beta (21–30 Hz)	0.13 (0.35)	0.14 (0.54)	0.01					
Anterior cingulate, 250–600 ms								
Theta	0.48 (0.80)	0.03 (0.83)	5.19*	5.01*	5.87*	4.62*	3.35	5.53*
Alpha	0.36 (0.76)	−0.11 (0.76)	6.45*	5.42*	6.40*	6.76*	2.68	7.42**
Low beta	0.06 (0.67)	−0.34 (0.52)	8.15**	7.41**	5.95*	9.23**	5.42*	9.18**
High beta	0.12 (0.80)	−0.23 (0.55)	4.93*	5.24*	6.10*	5.63*	3.93*	5.14*

Note. *P < 0.05, **P < 0.01.

CTD, chronic tic disorder; CYBOCS, Child Yale-Brown Obsessive-Compulsive Scale; dB, decibels; HC, healthy control; Hz, hertz; IQ, intelligence estimate; M, mean; SD, standard deviation; SWAN, Strengths and Weaknesses in ADHD Symptoms and Normal Behavior Scale.

### Oscillatory dynamics—clustering solution

**Figure 3:**
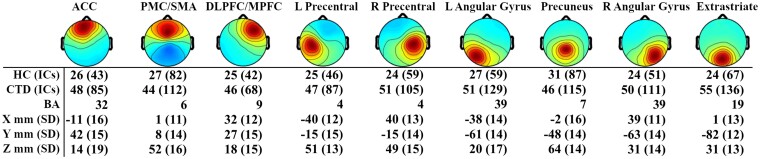
**Source-level independent component cluster topographies.** Scalp map views of propagated activity from the nine underlying source-level clusters included in the spectral power analysis. Number of subjects and independent components (ICs) within each cluster are listed for healthy control (HC) and chronic tic disorder (CTD) groups, as well as corresponding Brodmann Area (BA). Coordinates reflect cluster centroid with standard deviation of locations for all ICs in that cluster. ACC, anterior cingulate cortex; DLPFC, dorsolateral prefrontal cortex; L, left; MPFC, medial prefrontal cortex; PMC, premotor cortex; R, right; SD, standard deviation; SMA, supplementary motor area.

From the 12-cluster solution, three clusters (left temporal, right temporal and inferior occipital) were excluded after using 70% unique subject criteria. The resulting nine clusters and their corresponding scalp topographies are described in Fig. 3. Montreal Neurological Institute (MNI) coordinates of cluster centroids were used to estimate Brodmann areas for each cluster via the Yale BioImage Suite.^67^

### Spectral power during inhibitory control

**Figure 4:**
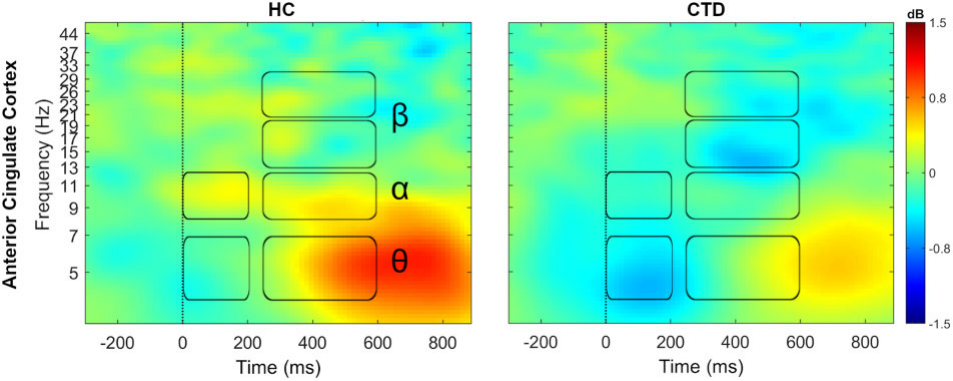
**Attenuated ACC power in chronic tic disorder.** During incongruent trials, subjects with chronic tic disorder (CTD) showed broadband attenuation in spectral power in the anterior cingulate cortex. Time-frequency ranges of significant between-group differences are marked by boxes for theta (θ, 4–7 Hz), alpha (α, 8–12 Hz), and low (13–20 Hz) and high beta (β, 20–30 Hz) frequency bands.

Oscillatory power during flanker performance was examined in the nine clusters selected for analysis for three conditions: congruent trials with correct responses, incongruent trials with correct responses, and incongruent trials with incorrect responses. MANOVA results were significant only for correct incongruent flanker trials, suggesting that task difficulty and successful management of conflict were important factors for evoking neural activation patterns unique to affected individuals. These group differences were present specifically in the ACC cluster during both the 0–200 ms [Wilk’s Λ = 0.86, *F*(4,69) = 2.8, *P* = 0.03] and 250–600 ms [Wilk’s Λ = 0.85, *F*(4,69) = 3.0, *P* = 0.03] time periods (Table 1; Fig. 4). Univariate ANOVAs revealed that directly after incongruent flanker presentation (0–200 ms), individuals with CTD exhibited attenuated theta [*F*(1,72)=4.2, *P* = 0.04] and alpha [*F*(1,72) = 10.1, *P* = 0.002] band power in the ACC relative to controls. In the subsequent 250–600 ms period, the HC group exhibited greater ACC spectral power compared to the CTD group in theta [*F*(1,72) = 5.2, *P* = 0.03], alpha [*F*(1,72) = 6.5, *P* = 0.01], low beta [*F*(1,72) = 8.1, *P* = 0.006], and high beta [*F*(1,72) = 4.9, *P* = 0.03]. Group differences remained significant after controlling for gender, age, and IQ, with the exception of ACC theta power during the 0–200 ms period, which was reduced to a trend level finding.

Controlling for age, partial correlations among spectral power, task performance and clinical measures indicated significant negative association between early (0–200 ms) alpha power with YGTSS Impairment (*r* = −0.31, *P* = 0.03) and later (250–600 ms) high beta power with incongruent trial accuracy (*r* = −0.25, *P* = 0.04). These results indicate that higher tic-related impairment and lower incongruent flanker accuracy were associated with EEG power modulations seen in CTD.

### Connectivity dynamics

Following findings of diagnostic group differences in spectral power exclusively on correct incongruent trials, effective connectivity was examined solely in this condition. In addition to whole-brain connectivity, we examined connectivity edges involving the ACC due to significant group differences in spectral power. Together, these two analyses revealed 10 connections (seven from the whole-brain analysis and three from the ACC analysis) with significant group differences in information flow. Each connection involved a minimum of 66 subjects using the 70% unique subject thresholding criteria. Notably, all connections were associated with bilateral precuneus or midcingulate cortex nodes, regions within the fronto-parietal network that have been shown to be highly involved in processes of cognitive control, integration of information, and mental imagery.^68^^,^^69^ These hubs showed differences in both information inflow and outflow with eight other anatomical regions located in mid-frontal, left motor, left temporal and bilateral occipital cortices.

**Figure 5:**
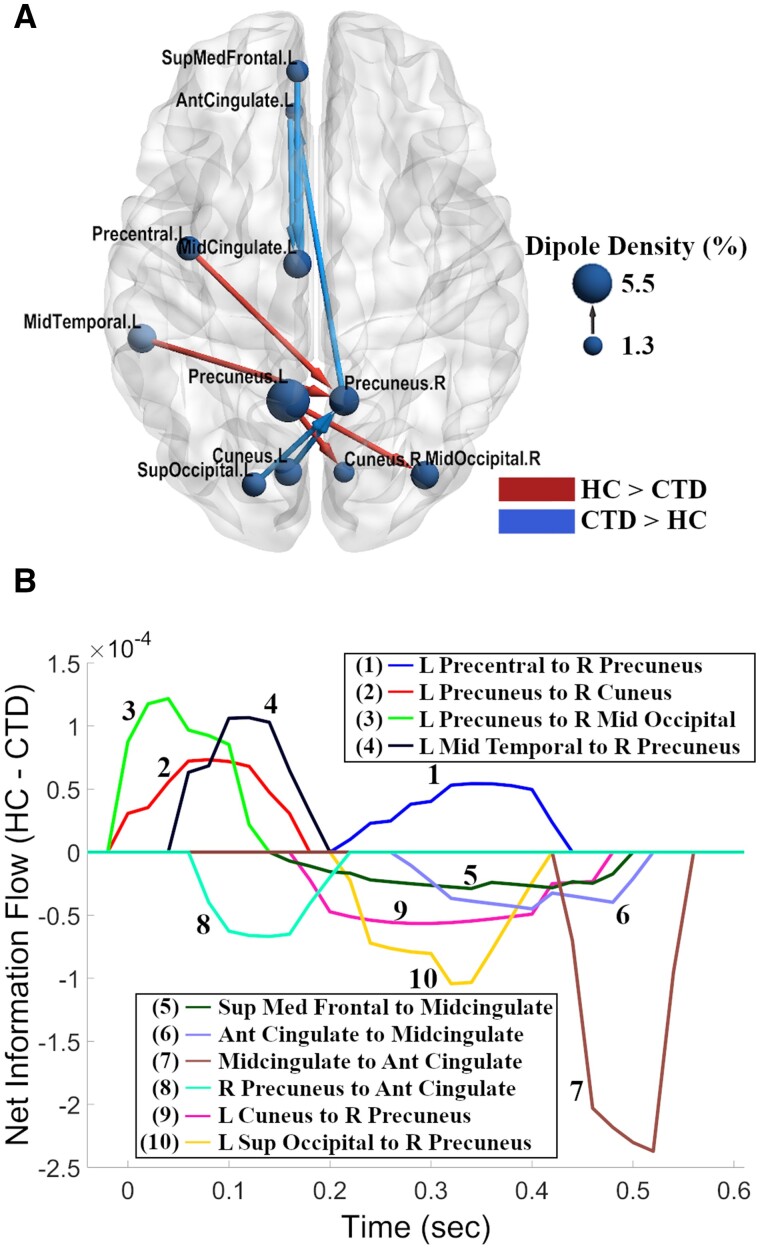
**Network view of effective connectivity during inhibitory control.** (**A**) The chronic tic disorder (CTD) group exhibited greater information flow along the midline and with frontal regions (blue), while greater causal flow among central and posterior regions was observed in the healthy control (HC) group (red). The arrows represent the direction of information flow for a given connection, and node sizes represent dipole density for a given node. The brain model was visualized using BrainNet Viewer software.^88^ (**B**) Time series representation of outflow for significant between-group connections from (**A**). Information flow which is stronger in the HC group is represented by positive values while stronger in the CTD group is represented by negative values. Stimulus onset was at *t* = 0; mean response time was ∼600 ms, averaged across both groups. Ant, anterior; L, left; Med, medial, R, right; Sup, superior.

Topographically, controls exhibited greater information flow within the central and posterior brain regions through integration of the precuneus with left precentral, left mid-temporal, and right occipital cortical sources (Fig. 5A). In contrast, individuals with CTD exhibited stronger effective connectivity along the midline and with anterior frontal areas, including the ACC and superior medial frontal cortex. Examination of the time dynamics of group differences revealed that differences in information flow occurred throughout the period from stimulus presentation until subject response (Fig. 5B). The HC group, however, displayed a notable period of relatively stronger information flow directly after stimulus presentation.

**Figure 6:**
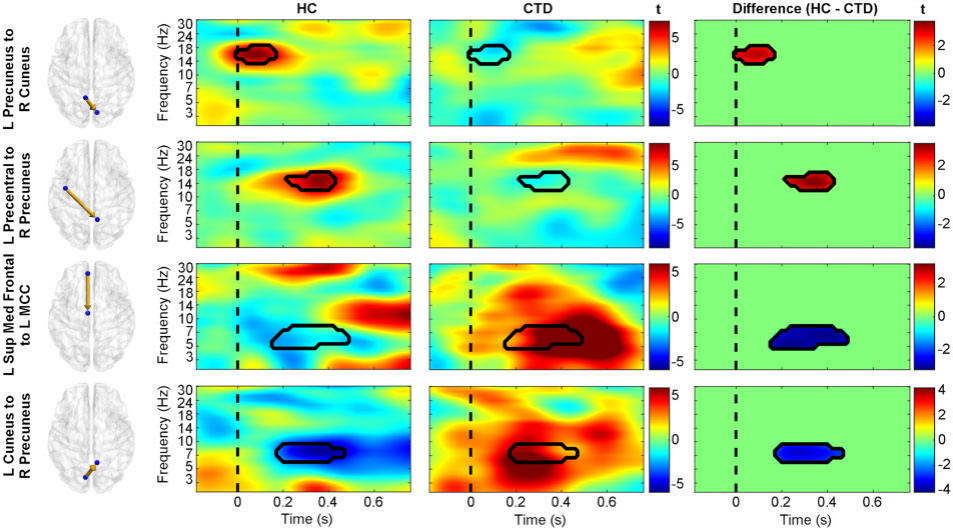
**Diagnostic group differences in causal information flow.** Time-frequency plots of effective connectivity patterns (represented as *t*-statistics) for healthy controls (HC, left), individuals with chronic tic disorder (CTD, middle), and the between-group differences (right). The precuneus in the HC group displayed greater connectivity with the right occipital and left precentral gyrus (red in difference plots), while the CTD group showed greater information flow with frontal and left occipital cortices (blue in difference plots). The dashed line represents stimulus presentation at *t* = 0. L, left; Med, medial; MCC, midcingulate cortex; R, right; Sup, superior.

**Figure 7:**
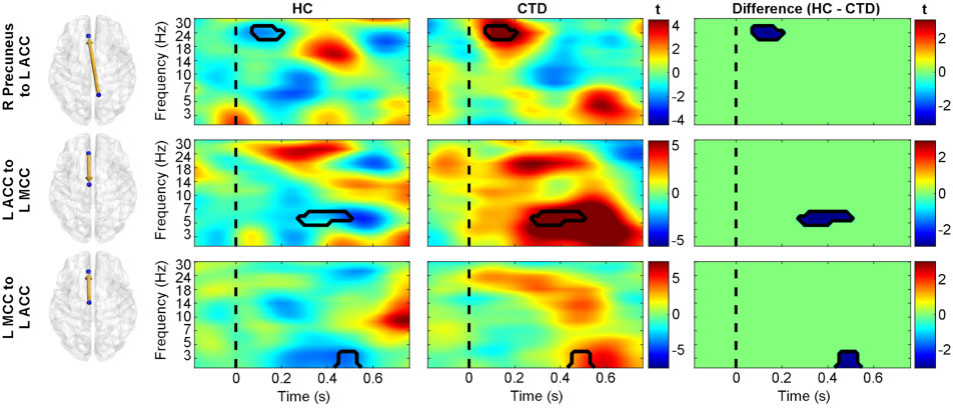
**Significant causal interactions involving the anterior cingulate.** Time-frequency plots of effective connectivity patterns (represented as *t*-statistics) for healthy controls (HC, left), individuals with chronic tic disorder (CTD, middle), and the between-group differences (right). Individuals with CTD exhibited greater effective connectivity (blue in difference plots) from the right precuneus to anterior cingulate cortex (ACC), as well as bidirectionally between the ACC and midcingulate cortex (MCC). The dashed line represents stimulus presentation at *t* = 0. L, left; R, right.

Controls exhibited greater information flow from the left precentral gyrus to right precuneus from 200 to 400 ms, revealing the first appearance of motor cortex differences in the study and suggesting atypical motor signalling from this area in individuals with CTD (Fig. 6). While controls displayed greater early left precuneus to right occipital communication, the CTD group showed stronger early connectivity from the right precuneus to ACC (Fig. 7), possibly representing different stimulus appraisal strategies. Recruitment of additional frontal regions for task processing in CTD was further indicated by greater information flow from the superior medial frontal cortex to midcingulate (Fig. 6), as well as greater bidirectional connectivity between the ACC and left midcingulate (Fig. 7). Given their timing (200–500 ms), these connections suggest atypically greater signalling of frontal conflict processing/response preparation mechanisms in individuals with CTD.

Partial correlations (controlling for age) between rPDC information flow and incongruent trial performance metrics indicated significant associations with several connections (*P* < 0.05). Accuracy was negatively correlated with left precentral to right precuneus (*r* = −0.27, *P* = 0.03) connectivity (which was stronger in controls) as well as with frontal connections (all of which were stronger in CTD) between the left superior medial frontal and midcingulate (*r* = −0.25, *P* = 0.04), ACC to midcingulate (*r* = −0.35, *P* = 0.003), midcingulate to ACC (*r* = −0.42, *P* = 0.0004), and right precuneus to ACC (*r* = −0.42, *P* = 0.0008). Midcingulate to ACC connectivity was also negatively correlated with incongruent trial reaction time (*r* = −0.31, *P* = 0.009). Correlations between ACC connectivity and spectral power were not significant (all *P*’s > 0.05), suggesting: (i) these are separate neural mechanisms that are not resultant from one another, and (ii) higher connectivity between frontal and midcingulate regions was an alternative neural pathway utilized by individuals with CTD that resulted in faster response times but similar accuracy.

## Discussion

The present study provides the first report of cortical source-resolved, event-related brain oscillatory dynamics and effective connectivity during inhibitory processing in CTD. Overall, children with CTD exhibited lower spectral power in the ACC but higher causal information flow between the ACC and other midline central and posterior regions, despite similar levels of task accuracy, relative to typically developing controls. In addition, whole-brain connectivity analyses indicated that the midcingulate and precuneus serve as fronto-parietal network hubs whose connections with several motor and sensory areas such as left precentral, left temporal, and bilateral occipital nodes were atypical in CTD. Finally, attenuated ACC activation and higher information flow between and among nodes within the fronto-parietal attention network were significantly associated with tic impairment, faster reaction time, and worse inhibitory performance, suggesting these alternate cortical patterns may be a putative neural adaptation among individuals with CTD.

Significant differences in neural dynamics were observed between controls and individuals with CTD despite equivocal differences in inhibitory task performance, a finding that is consistent with other event-related potential and functional MRI cognitive control studies.^14^^,^^15^^,^^70^ Interestingly, these differences in dynamics were observed solely in incongruent trials despite the group differences present in congruent trial reaction time. A potential explanation for this may be found from the correlation analyses, which revealed significant EEG correlations primarily with incongruent trial accuracy. This could be due to a greater sensitivity of the EEG measurements used to neural mechanisms associated with accuracy rather than network activity related to reaction time, which may be widely distributed.

Within the current study, the ACC showed between-group differences in both activation and connectivity patterns, suggesting it plays a key role in the cognitive inhibition process. The involvement of this region is well supported, as the ACC has repeatedly been shown to be engaged during monitoring of conflict.^71–73^ In CTD, studies have reported structural and neurochemical ACC deficits among affected individuals, including lower grey matter in adults^74^ and lower γ-aminobutyric acid (GABA) levels in youth.^75^ Aberrant ACC activity has also been observed in paediatric and adult samples during inhibitory control tasks^15^^,^^76^ as well as prior to tic occurrence,^77^ suggesting the ACC subserves multiple roles common to both tic occurrence and inhibitory control in CTD. This hypothesis is further supported by negative correlations observed here between alpha band power in the ACC and higher YGTSS Impairment scores, indicating greater tic impairment was associated with attenuated ACC activity during inhibitory processing.

Several additional brain regions exhibited aberrant effective connectivity, suggesting widespread atypical network communication in individuals with CTD during inhibitory control. The precuneus and midcingulate were involved with nearly all significant connections. Both regions are known to be associated with the integration of various processing areas and cortical functions, including cognitive control,^69^^,^^72^^,^^78^ and have been implicated in CTD during an adaptive control paradigm.^79^ The precuneus communicates with peripheral visual, frontal and motor regions,^68^ and the presence of altered hub connectivity in CTD may indicate difficulties with the integration of multimodal information that is required when performing sensory-driven movements. The precuneus is also associated with internally guided attention^78^ as well as with attention shifting between object features.^80^ Given that the paradigm was designed for assessing features of attentional control,^81^ altered connectivity patterns between the precuneus and visual cortex could suggest mechanistic and/or procedural differences in attention shifting between important stimulus features (e.g. arrow directionality) in CTD, as reflected by contrasting directions of information flow between the precuneus and bilateral occipital cortices by diagnostic group. Together these would suggest individuals with CTD utilize atypical communication patterns between attention, visual and sensorimotor networks.

While the involvement of the MCC in cognitive control and response selection is well established, greater engagement of the midcingulate with other frontal areas could provide support for an urge-based motoric role that has been previously hypothesized.^82^ In particular, this hypothesis proposes a separation between a ‘urge-driven action’ network involving the insula and MCC and a ‘willed, intentional action’ network primarily involving the premotor and parietal cortices. The present findings fit well with this anatomical separation, as while a premotor and parietal-based connectivity pattern was observed in controls, an additional MCC-based network was present in the CTD group. This idea is also supported by the fact that negative, rather than positive, correlations were observed between MCC connectivity and accuracy as well as reaction time, indicating that the influence of the MCC did not necessarily help participants perform better on the task. Additionally, the involvement of the MCC in goal-directed (including impulsive and body-directed) actions^83^ could signify greater impulsive signalling within these motor pathways in CTD and help explain the EEG–behaviour relationships.

Whole brain effective connectivity analyses revealed greater information flow among the central and posterior network hubs in the HC group, while the CTD group exhibited greater connectivity along the midline fronto-parietal axis during inhibitory processing. Greater fronto-parietal connectivity is typically considered to be a developmentally mature neural pattern,^29^ which may arise among children with CTD as a result of frequent inhibition of tics and the need to control tic expression.^19^^,^^34^ On the other hand, integration of the ACC into the fronto-parietal network as opposed to the cingulo-opercular control network is an immature developmental pattern observed in youth with CTD that is most commonly seen among typically developing children but not adolescents or adults.^29^ Thus, these results suggest altered network connectivity patterns that cannot be characterized as unidirectional along the developmental spectrum.

While qualitatively different developmental trajectories of neural connectivity have been reported previously,^34^ we note that another reason these results don’t map directly onto previous findings may be the lack of comparable studies given the dearth of task dependent, event-related connectivity studies in CTD. Many MRI-based studies reporting functional or structural connectivity have been performed on resting state, which may differ significantly from task dependent effective connectivity due to increased top down control of cortico-striatal-thalamic activity under task conditions.^84^ While several studies have examined connectivity dynamics during tic suppression and other inhibitory control paradigms,^85^ of the two EEG-based studies examining scalp-based connectivity, children with CTD exhibited higher coherence values relative to controls in fronto-central connections during tic suppression^39^ and motor inhibition.^38^ Our results are thus consistent with these previous studies and extend the findings with a higher density electrode montage and cortical-source level effective connectivity, allowing identification of directional information flow to specific nodes along the fronto-parietal network with precise timing. In light of these associations, altered task-dependent network connectivity patterns within CTD appear to have greater involvement of frontal regions than is generally reported for typically developing children. We hypothesize that greater frontal connectivity is likely a neural adaptation to coping with the illness, but one that does not necessarily result in better behavioural performance.

A strength of this study is the large sample size of children with and without CTDs (*N* = 95) who are within a relatively tight age range of 8–12 years. In addition, state of the art EEG recording and processing techniques allowed for sub-second quantification of oscillatory activity and effective connectivity with greater spatial resolution of cortical-source resolved generators. One limitation is that paediatric populations are developmentally heterogenous and at the early stages of diagnosis, which may make it difficult to generalize findings towards older individuals with tic disorder. Nevertheless, evaluations of early diagnostic stages are prudent for examining disorder progression and optimizing early intervention techniques. A second limitation is that EEG is blind to activity from deeper subcortical sources within the cortico-striato-thalamo-cortical network, which are thought to play a key role in tic generation within CTDs.^86^^,^^87^ Key distinctions have been made, however, between the neural mechanisms that generate tics versus those that play a role in suppression of involuntary and voluntary movements, as well as inhibition of internally driven versus externally triggered events.^19^ Recent studies and a meta-analysis of task-based neuroimaging studies did not find significant diagnostic group differences in activation and network disruptions involving basal ganglia or thalamic regions,^24^^,^^26^^,^^27^ suggesting there are clear cortical dysfunctions that contribute meaningfully to CTD phenomenology that can be assessed with EEG. Furthermore, the atypical cortical activity reported here may provide potential non-invasive neuromodulation targets for future treatment of CTD. A final consideration is that the CTD sample contained more boys and some subjects with comorbid OCD and ADHD. The occurrence of comorbid disorders in patients with CTD is typical but creates potential issues when attempting to interpret results and their association with CTD. However, most group differences remained significant after controlling for sex as well as ADHD and OCD symptoms, suggesting that the group differences observed are not systematically associated with gender or diagnostic comorbidity. In summary, we have provided evidence of aberrant activation and communication patterns within the fronto-parietal network, specifically in the anterior cingulate cortex, which had attenuated spectral power but greater causal information flow with several fronto-parietal hub regions, particularly the midcingulate and precuneus, in CTD. Further research into these temporally resolved mechanisms will better elucidate neural mechanisms and potential patterns of atypical network communication that lead to tic expression and suppression in CTD.

## Abbreviations

ACC =: anterior cingulate cortex
ADHD =: attention deficit/hyperactivity disorder
CTD =: chronic tic disorder
ERSP =: event-related spectral perturbation
HC =: healthy controls
OCD =: obsessive compulsive disorder
rPDC =: renormalized partial directed coherence
YGTSS =: Yale Global Tic Severity Scale

## References

[1] Ozonoff S , Strayer DL, McMahon WM, Filloux F. Inhibitory deficits in Tourette syndrome: A function of comorbidity and symptom severity. J Child Psychol Psychiatry. 1998;39(8):1109–1118. 9844981

[2] Roessner V , Albrecht B, Dechent P, Baudewig J, Rothenberger A. Normal response inhibition in boys with Tourette syndrome. Behav Brain Funct. 2008;4(1):29. 18638368 10.1186/1744-9081-4-29 PMC2491645

[3] Crawford S , Channon S, Robertson MM. Tourette’s syndrome: Performance on tests of behavioural inhibition, working memory and gambling. J Child Psychol Psychiatry. 2005;46(12):1327–1336. 16313433 10.1111/j.1469-7610.2005.01419.x

[4] Wylie SA , Claassen DO, Kanoff KE, Ridderinkhof KR, van den Wildenberg WPM. Impaired inhibition of prepotent motor actions in patients with Tourette syndrome. J Psychiatry Neurosci. 2013;38(5):349–356. 23820185 10.1503/jpn.120138 PMC3756119

[5] Mueller SC , Jackson GM, Dhalla R, Datsopoulos S, Hollis CP. Enhanced cognitive control in young people with Tourette’s syndrome. Curr Biol. 2006;16(6):570–573. 16546080 10.1016/j.cub.2006.01.064

[6] Jackson SR , Parkinson A, Jung J, et al Compensatory neural reorganization in Tourette syndrome. Curr Biol. 2011;21(7):580–585. 21439830 10.1016/j.cub.2011.02.047 PMC3076629

[7] Morand-Beaulieu S , Grot S, Lavoie J, Leclerc JB, Luck D, Lavoie ME. The puzzling question of inhibitory control in Tourette syndrome: A meta-analysis. Neurosci Biobehav Rev. 2017;80:240–262. 28502600 10.1016/j.neubiorev.2017.05.006

[8] Eriksen BA , Eriksen CW. Effects of noise letters upon the identification of a target letter in a nonsearch task. Percept Psychophys. 1974;16(1):143–149.

[9] Fan J , Mccandliss B, Fossella J, Flombaum J, Posner M. The activation of attentional networks. NeuroImage. 2005;26(2):471–479. 15907304 10.1016/j.neuroimage.2005.02.004

[10] Fan J , Byrne J, Worden MS, et al The relation of brain oscillations to attentional networks. J Neurosci off J Soc Neurosci. 2007;27(23):6197–6206. 10.1523/JNEUROSCI.1833-07.2007 PMC6672149 17553991

[11] Xuan B , Mackie M-A, Spagna A, et al The activation of interactive attentional networks. Neuroimage. 2016;129:308–319. 26794640 10.1016/j.neuroimage.2016.01.017 PMC4803523

[12] Bunge SA , Dudukovic NM, Thomason ME, Vaidya CJ, Gabrieli JDE. Immature frontal lobe contributions to cognitive control in children: Evidence from fMRI. Neuron. 2002;33(2):301–311. 11804576 10.1016/s0896-6273(01)00583-9 PMC4535916

[13] Santhana Gopalan PR , Loberg O, Hämäläinen JA, Leppänen PHT. Attentional processes in typically developing children as revealed using brain event-related potentials and their source localization in Attention Network Test. Sci Rep. 2019;9(1):2940. 30814533 10.1038/s41598-018-36947-3 PMC6393460

[14] Baym CL , Corbett BA, Wright SB, Bunge SA. Neural correlates of tic severity and cognitive control in children with Tourette syndrome. Brain. 2008;131(Pt 1):165–179. 18056159 10.1093/brain/awm278

[15] Jung J , Jackson SR, Parkinson A, Jackson GM. Cognitive control over motor output in Tourette syndrome. Neurosci Biobehav Rev. 2013;37(6):1016–1025. 23017869 10.1016/j.neubiorev.2012.08.009

[16] Ganos C , Kahl U, Brandt V, et al The neural correlates of tic inhibition in Gilles de la Tourette syndrome. Neuropsychologia. 2014;65:297–301. 25128587 10.1016/j.neuropsychologia.2014.08.007

[17] Fan S , Cath DC, van der Werf YD, et al Trans-diagnostic comparison of response inhibition in Tourette’s disorder and obsessive-compulsive disorder. World J Biol Psychiatry. 2018;19(7):527–537. 28741401 10.1080/15622975.2017.1347711

[18] Tinaz S , Belluscio BA, Malone P, van der Veen JW, Hallett M, Horovitz SG. Role of the sensorimotor cortex in tourette syndrome using multimodal imaging. Hum Brain Mapp. 2014;35(12):5834–5846. 25044024 10.1002/hbm.22588 PMC4776755

[19] Ganos C , Rothwell J, Haggard P. Voluntary inhibitory motor control over involuntary tic movements. Mov Disord. 2018;33(6):937–946. 29508917 10.1002/mds.27346

[20] Loo SK , Miyakoshi M, Tung K, et al Neural activation and connectivity during cued eye blinks in chronic tic disorders. NeuroImage Clin. 2019;24:101956 31382238 10.1016/j.nicl.2019.101956 PMC6698693

[21] Zapparoli L , Macerollo A, Joyce EM, Martino D, Kilner JM. Voluntary tic suppression and the normalization of motor cortical beta power in Gilles de la Tourette syndrome: An EEG study. Eur J Neurosci. 2019;50(12):3944–3957. 31421054 10.1111/ejn.14548

[22] Fan S , van den Heuvel OA, Cath DC, et al Altered functional connectivity in resting state networks in Tourette’s disorder. Front Hum Neurosci. 2018;12:363. 30279651 10.3389/fnhum.2018.00363 PMC6154258

[23] Cui Y , Jin Z, Chen X, He Y, Liang X, Zheng Y. Abnormal baseline brain activity in drug-naïve patients with Tourette syndrome: A resting-state fMRI study. Front Hum Neurosci. 2014;7:913 24427134 10.3389/fnhum.2013.00913 PMC3877773

[24] Wen H , Liu Y, Wang J, et al Combining tract- and atlas-based analysis reveals microstructural abnormalities in early Tourette syndrome children. Hum Brain Mapp. 2016;37(5):1903–1919. 26929221 10.1002/hbm.23146 PMC6867590

[25] Hashemiyoon R , Kuhn J, Visser-Vandewalle V. Putting the pieces together in Gilles de la Tourette syndrome: Exploring the link between clinical observations and the biological basis of dysfunction. Brain Topogr. 2017;30(1):3–29. 27783238 10.1007/s10548-016-0525-z PMC5219042

[26] Polyanska L , Critchley HD, Rae CL. Centrality of prefrontal and motor preparation cortices to Tourette syndrome revealed by meta-analysis of task-based neuroimaging studies. Neuroimage Clin. 2017;16:257–267. 28831377 10.1016/j.nicl.2017.08.004 PMC5554925

[27] Wen H , Liu Y, Rekik I, et al Disrupted topological organization of structural networks revealed by probabilistic diffusion tractography in Tourette syndrome children. Hum Brain Mapp. 2017;38(8):3988–4008. 28474385 10.1002/hbm.23643 PMC6866946

[28] Openneer TJC , Marsman J-BC, van der Meer D, et al A graph theory study of resting-state functional connectivity in children with Tourette syndrome. Cortex. 2020;126:63–72. 32062470 10.1016/j.cortex.2020.01.006

[29] Church JA , Fair DA, Dosenbach NUF, et al Control networks in paediatric Tourette syndrome show immature and anomalous patterns of functional connectivity. Brain. 2009;132(Pt 1):225–238. 18952678 10.1093/brain/awn223 PMC2638693

[30] Ramkiran S , Heidemeyer L, Gaebler A, Shah NJ, Neuner I. Alterations in basal ganglia-cerebello-thalamo-cortical connectivity and whole brain functional network topology in Tourette’s syndrome. Neuroimage Clin. 2019;24:101998. 31518769 10.1016/j.nicl.2019.101998 PMC6742843

[31] Worbe Y , Marrakchi-Kacem L, Lecomte S, et al Altered structural connectivity of cortico-striato-pallido-thalamic networks in Gilles de la Tourette syndrome. Brain. 2015;138(Pt 2):472–482. 25392196 10.1093/brain/awu311 PMC4306818

[32] Worbe Y , Malherbe C, Hartmann A, et al Functional immaturity of cortico-basal ganglia networks in Gilles de la Tourette syndrome. Brain. 2012;135(Pt 6):1937–1946. 22434213 10.1093/brain/aws056

[33] Tinaz S , Malone P, Hallett M, Horovitz SG. Role of the right dorsal anterior insula in the urge to tic in tourette syndrome. Mov Disord. 2015;30(9):1190–1197. 25855089 10.1002/mds.26230 PMC5088605

[34] Nielsen AN , Gratton C, Church JA, et al Atypical functional connectivity in Tourette syndrome differs between children and adults. Biol Psychiatry. 2020;87(2):164–173. 31472979 10.1016/j.biopsych.2019.06.021 PMC6925331

[35] Franzkowiak S , Pollok B, Biermann-Ruben K, et al Motor-cortical interaction in Gilles de la Tourette syndrome. PLoS One. 2012;7(1):e27850. 22238571 10.1371/journal.pone.0027850 PMC3251574

[36] Atkinson-Clement C , Porte C-A, de Liege A, et al Impulsive prepotent actions and tics in Tourette disorder underpinned by a common neural network. Mol Psychiatry. 2020;29:1–10. 10.1038/s41380-020-00890-5 PMC8505252 32994553

[37] Rae CL , Polyanska L, Gould van Praag CD, et al Face perception enhances insula and motor network reactivity in Tourette syndrome. Brain. 2018;141(11):3249–3261. 30346484 10.1093/brain/awy254 PMC6202569

[38] Serrien DJ , Orth M, Evans AH, Lees AJ, Brown P. Motor inhibition in patients with Gilles de la Tourette syndrome: Functional activation patterns as revealed by EEG coherence. Brain. 2005;128(Pt 1):116–125. 15496435 10.1093/brain/awh318

[39] Hong HJ , Sohn H, Cha M, et al Increased frontomotor oscillations during tic suppression in children with Tourette syndrome. J Child Neurol. 2013;28(5):615–624. 22859696 10.1177/0883073812450317

[40] Nunez PL , Srinivasan R, Westdorp AF, et al EEG coherency: I: Statistics, reference electrode, volume conduction, Laplacians, cortical imaging, and interpretation at multiple scales. Electroencephalogr Clin Neurophysiol. 1997;103(5):499–515. 9402881 10.1016/s0013-4694(97)00066-7

[41] Koshiyama D , Miyakoshi M, Joshi YB, et al Abnormal effective connectivity underlying auditory mismatch negativity impairments in schizophrenia. Biol Psychiatry Cogn Neurosci Neuroimaging. 2020;5(11):1028–1039. 32830097 10.1016/j.bpsc.2020.05.011

[42] Silverman WK. Anxiety disorders interview schedule for DSM-IV: Parent interview schedule. New York, NY: Oxford University Press; 1996.

[43] Leckman JF , Riddle MA, Hardin MT, et al The Yale Global Tic Severity Scale: Initial testing of a clinician-rated scale of tic severity. J Am Acad Child Adolesc Psychiatry. 1989;28(4):566–573. 2768151 10.1097/00004583-198907000-00015

[44] Scahill L , Riddle MA, McSwiggin-Hardin M, et al Children’s Yale-Brown Obsessive Compulsive Scale: Reliability and validity. J Am Acad Child Adolesc Psychiatry. 1997;36(6):844–852. 9183141 10.1097/00004583-199706000-00023

[45] Swanson JM , Schuck S, Porter MM, et al Categorical and dimensional definitions and evaluations of symptoms of ADHD: History of the SNAP and the SWAN Rating Scales. Int J Educ Psychol Assess. 2012;10(1):51–70. 26504617 PMC4618695

[46] Wechsler D. Wechsler abbreviated scale of intelligence WASI: Manual. San Antonio, TX: Pearson/PsychCorpl; 1999.

[47] Delorme A , Makeig S. EEGLAB: An open source toolbox for analysis of single-trial EEG dynamics including independent component analysis. J Neurosci Methods. 2004;134(1):9–21. 15102499 10.1016/j.jneumeth.2003.10.009

[48] Kothe CA , Makeig S. BCILAB: A platform for brain–computer interface development. J Neural Eng. 2013;10(5):056014. 23985960 10.1088/1741-2560/10/5/056014

[49] Mullen TR , Kothe CAE, Chi YM, et al Real-time neuroimaging and cognitive monitoring using wearable dry EEG. IEEE Trans Biomed Eng. 2015;62(11):2553–2567. 26415149 10.1109/TBME.2015.2481482 PMC4710679

[50] Chang C-Y , Hsu S-H, Pion-Tonachini L, Jung T-P. Evaluation of artifact subspace reconstruction for automatic EEG artifact removal. Annu Int Conf IEEE Eng Med Biol Soc . 2018;2018:1242–1245. 30440615 10.1109/EMBC.2018.8512547

[51] Blum S , Jacobsen NSJ, Bleichner MG, Debener S. A Riemannian modification of artifact subspace reconstruction for EEG artifact handling. Front Hum Neurosci. 2019;13:141- 31105543 10.3389/fnhum.2019.00141 PMC6499032

[52] Palmer JA , Makeig S, Kreutz-Delgado K, Rao BD. Newton method for the ICA mixture model. In: 2008 IEEE International Conference on Acoustics, Speech and Signal Processing. IEEE. 2008:1805–1808.

[53] Hsu S-H , Pion-Tonachini L, Palmer J, Miyakoshi M, Makeig S, Jung T-P. Modeling brain dynamic state changes with adaptive mixture independent component analysis. NeuroImage. 2018;183:47–61. 30086409 10.1016/j.neuroimage.2018.08.001 PMC6205696

[54] Delorme A , Palmer J, Onton J, Oostenveld R, Makeig S. Independent EEG sources are dipolar. PLoS One. 2012;7(2):e30135. 22355308 10.1371/journal.pone.0030135 PMC3280242

[55] Pion-Tonachini L , Makeig S, Kreutz-Delgado K. Crowd labeling latent Dirichlet allocation. Knowl Inf Syst. 2017;53(3):749–765. 30416242 10.1007/s10115-017-1053-1 PMC6223327

[56] Oostenveld R , Fries P, Maris E, Schoffelen J-M. FieldTrip: Open source software for advanced analysis of MEG, EEG, and invasive electrophysiological data. Comput Intell Neurosci. 2010;2011:e156869. 10.1155/2011/156869 PMC3021840 21253357

[57] Korzeniewska A , Crainiceanu CM, Kuś R, Franaszczuk PJ, Crone NE. Dynamics of event-related causality in brain electrical activity. Hum Brain Mapp. 2008;29(10):1170–1192. 17712784 10.1002/hbm.20458 PMC6870676

[58] Delorme A , Mullen T, Kothe C, et al EEGLAB, SIFT, NFT, BCILAB, and ERICA: New tools for advanced EEG processing. Comput Intell Neurosci. 2011;2011:130714–130712. 21687590 10.1155/2011/130714 PMC3114412

[59] Schelter B , Timmer J, Eichler M. Assessing the strength of directed influences among neural signals using renormalized partial directed coherence. J Neurosci Methods. 2009;179(1):121–130. 19428518 10.1016/j.jneumeth.2009.01.006

[60] Tzourio-Mazoyer N , Landeau B, Papathanassiou D, et al Automated anatomical labeling of activations in SPM using a macroscopic anatomical parcellation of the MNI MRI single-subject brain. Neuroimage. 2002;15(1):273–289. 11771995 10.1006/nimg.2001.0978

[61] Korn EL , Troendle JF, McShane LM, Simon R. Controlling the number of false discoveries: Application to high-dimensional genomic data. J Stat Plan Inference. 2004;124(2):379–398.

[62] Groppe DM , Urbach TP, Kutas M. Mass univariate analysis of event-related brain potentials/fields I: A critical tutorial review. Psychophysiology. 2011;48(12):1711–1725. 21895683 10.1111/j.1469-8986.2011.01273.x PMC4060794

[63] R Core Team. R: A language and environment for statistical computing. Vienna, Austria: R Foundation for Statistical Computing; 2020. http://www.R-project.org

[64] Cohen J. Statistical power analysis for the behavioral sciences. Mahwah, NJ: Academic Press; 1988.

[65] Debes NMMM , Lange T, Jessen TL, Hjalgrim H, Skov L. Performance on Wechsler intelligence scales in children with Tourette syndrome. Eur J Paediatr Neurol. 2011;15(2):146–154. 20739206 10.1016/j.ejpn.2010.07.007

[66] Kumar A , Trescher W, Byler D. Tourette syndrome and comorbid neuropsychiatric conditions. Curr Dev Disord Rep. 2016;3(4):217–221. 27891299 10.1007/s40474-016-0099-1 PMC5104764

[67] Lacadie CM , Fulbright RK, Rajeevan N, Constable RT, Papademetris X. More accurate Talairach coordinates for neuroimaging using nonlinear registration. Neuroimage. 2008;42(2):717–725. 18572418 10.1016/j.neuroimage.2008.04.240 PMC2603575

[68] Margulies DS , Vincent JL, Kelly C, et al Precuneus shares intrinsic functional architecture in humans and monkeys. Proc Natl Acad Sci USA. 2009;106(47):20069–20074. 19903877 10.1073/pnas.0905314106 PMC2775700

[69] Shackman AJ , Salomons TV, Slagter HA, Fox AS, Winter JJ, Davidson RJ. The integration of negative affect, pain and cognitive control in the cingulate cortex. Nat Rev Neurosci. 2011;12(3):154–167. 21331082 10.1038/nrn2994 PMC3044650

[70] Ganos C , Kühn S, Kahl U, et al Action inhibition in Tourette syndrome. Mov Disord. 2014;29(12):1532–1538. 24995958 10.1002/mds.25944

[71] Van Veen V , Carter C. The anterior cingulate as a conflict monitor: fMRI and ERP studies. Physiol Behav. 2002;77(4–5):477–482. 12526986 10.1016/s0031-9384(02)00930-7

[72] Fan J , Flombaum JI, McCandliss BD, Thomas KM, Posner MI. Cognitive and brain consequences of conflict. Neuroimage. 2003;18(1):42–57. 12507442 10.1006/nimg.2002.1319

[73] Botvinick MM , Cohen JD, Carter CS. Conflict monitoring and anterior cingulate cortex: An update. Trends Cogn Sci. 2004;8(12):539–546. 15556023 10.1016/j.tics.2004.10.003

[74] Müller-Vahl KR , Kaufmann J, Grosskreutz J, Dengler R, Emrich HM, Peschel T. Prefrontal and anterior cingulate cortex abnormalities in Tourette syndrome: Evidence from voxel-based morphometry and magnetization transfer imaging. BMC Neurosci. 2009;10(1):47. 19435502 10.1186/1471-2202-10-47 PMC2691409

[75] Freed RD , Coffey BJ, Mao X, et al Decreased anterior cingulate cortex γ-aminobutyric acid in youth with Tourette’s disorder. Pediatr Neurol. 2016;65:64–70. 27743746 10.1016/j.pediatrneurol.2016.08.017 PMC13292813

[76] Marsh R , Zhu H, Wang Z, Skudlarski P, Peterson BS. A developmental fMRI study of self-regulatory control in Tourette’s syndrome. Am J Psychiatry. 2007;164(6):955–966. 17541057 10.1176/appi.ajp.164.6.955 PMC2291294

[77] Wang Z , Maia TV, Marsh R, Colibazzi T, Gerber A, Peterson BS. The neural circuits that generate tics in Tourette’s syndrome. Am J Psychiatry. 2011;168(12):1326–1337. 21955933 10.1176/appi.ajp.2011.09111692 PMC4246702

[78] Cavanna AE , Trimble MR. The precuneus: A review of its functional anatomy and behavioural correlates. Brain. 2006;129(Pt 3):564–583. 16399806 10.1093/brain/awl004

[79] Church J , Wenger KK, Dosenbach NUF, Miezin FM, Petersen SE, Schlaggar BL. Task control signals in pediatric Tourette syndrome show evidence of immature and anomalous functional activity. Front Hum Neurosci. 2009;3:38. 19949483 10.3389/neuro.09.038.2009 PMC2784679

[80] Nagahama Y , Okada T, Katsumi Y, et al Transient neural activity in the medial superior frontal gyrus and precuneus time locked with attention shift between object features. Neuroimage. 1999;10(2):193–199. 10417251 10.1006/nimg.1999.0451

[81] Fan J , McCandliss BD, Sommer T, Raz A, Posner MI. Testing the efficiency and independence of attentional networks. J Cogn Neurosci. 2002;14(3):340–347. 11970796 10.1162/089892902317361886

[82] Jackson SR , Parkinson A, Kim SY, Schüermann M, Eickhoff SB. On the functional anatomy of the urge-for-action. Cogn Neurosci. 2011;2(3–4):227–243. 22299020 10.1080/17588928.2011.604717 PMC3259619

[83] Caruana F , Gerbella M, Avanzini P, et al Motor and emotional behaviours elicited by electrical stimulation of the human cingulate cortex. Brain. 2018;141(10):3035–3051. 30107501 10.1093/brain/awy219

[84] Zapparoli L , Tettamanti M, Porta M, et al A tug of war: Antagonistic effective connectivity patterns over the motor cortex and the severity of motor symptoms in Gilles de la Tourette syndrome. Eur J Neurosci. 2017;46(6):2203–2213. 28833746 10.1111/ejn.13658

[85] Zapparoli L , Porta M, Paulesu E. The anarchic brain in action: The contribution of task-based fMRI studies to the understanding of Gilles de la Tourette syndrome. Curr Opin Neurol. 2015;28(6):604–611. 26402403 10.1097/WCO.0000000000000261

[86] Ganos C , Roessner V, Münchau A. The functional anatomy of Gilles de la Tourette syndrome. Neurosci Biobehav Rev. 2013;37(6):1050–1062. 23237884 10.1016/j.neubiorev.2012.11.004

[87] Jackson GM , Draper A, Dyke K, Pépés SE, Jackson SR. Inhibition, disinhibition, and the control of action in Tourette syndrome. Trends Cogn Sci. 2015;19(11):655–665. 26440120 10.1016/j.tics.2015.08.006

[88] Xia M , Wang J, He Y. BrainNet Viewer: A network visualization tool for human brain connectomics. Csermely P, ed. PLoS One. 2013;8(7):e68910. 23861951 10.1371/journal.pone.0068910 PMC3701683

